# Cutting-edge novel device in the treatment of obesity

**DOI:** 10.1055/a-2209-3374

**Published:** 2024-01-30

**Authors:** Jan Kral, Jana Selucka, Katerina Waloszkova, Marek Buzga, Julius Spicak, Evzen Machytka

**Affiliations:** 1 Institute for Clinical and Experimental Medicine, Hepatogastroenterology Department, Prague, Czech Republic; 2 Department of Internal Medicine, Second Faculty of Medicine, Charles University, Prague, Czech Republic; 3 Department of Physiology and Pathophysiology, Faculty of Medicine, University of Ostrava, Ostrava, Czech Republic

A 57-year-old patient with arterial hypertension, dyslipidemia, and obesity grade I (BMI 34.9 kg/m ^2^ , 95 kg, 165 cm) underwent a new and unique sleeve gastroplasty procedure for weight reduction ( Video 1 ).

**Video 1** Endoscopic sleeve gastroplasty using a novel device.


A novel device is used to perform the sleeve gastroplasty ( Fig. 1 ). The specialized device is inserted into the patient through an overtube, and a thin nasal endoscope is passed through the device into the stomach. Under visual guidance, the appropriate position in the stomach (antrum) is identified, and a vacuum (–84 kPa) is initiated. Subsequently, the nasal endoscope is removed, and the device channel is closed to maintain the vacuum. In this phase of the procedure, full-thickness suturing of the gastric wall is performed using a circular stitch ( Fig. 2 ). The device automatically terminates the stitch, and it is withdrawn through the overtube. The sutures are then inspected using an endoscope. This process is repeated 4–5 times, depending on the appearance of the sleeve gastroplasty. After completing all 4–5 sutures, the resulting sleeve is inspected, including checking for possible complications such as bleeding or perforation. The procedure is performed under general anesthesia, with observation continuing until the following day. The average duration of the procedure is 40 minutes.

**Fig. 1:**
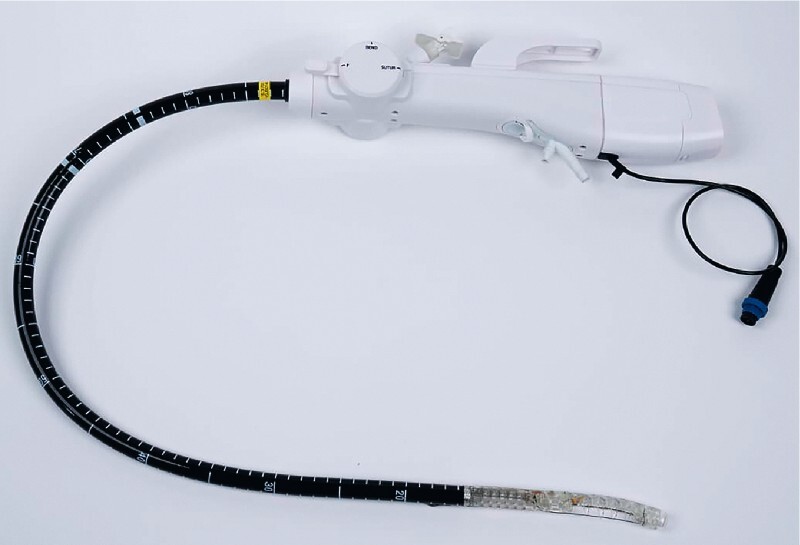
Device overview.

**Fig. 2:**
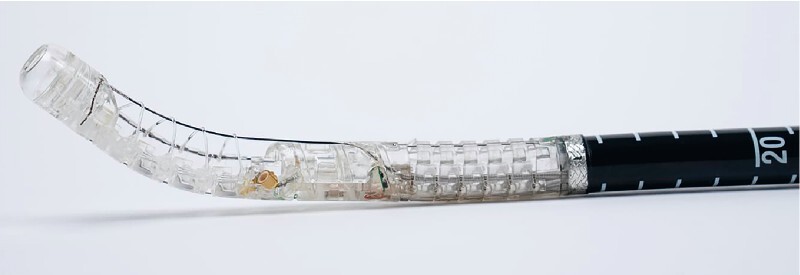
Assembled device.

After three months, the patient achieved a weight loss of 8 kg (8.4 % total body weight loss) without any signs of complications. This novel device appears to be a promising new method for weight reduction that is fast, feasible, and safe ( Fig. 3 ). Randomized studies comparing its effectiveness to other devices intended for endoscopic sleeve gastroplasty, where the percentage of total body weight loss at 6 months averages around 15 %, are now needed 1 .

**Fig. 3:**
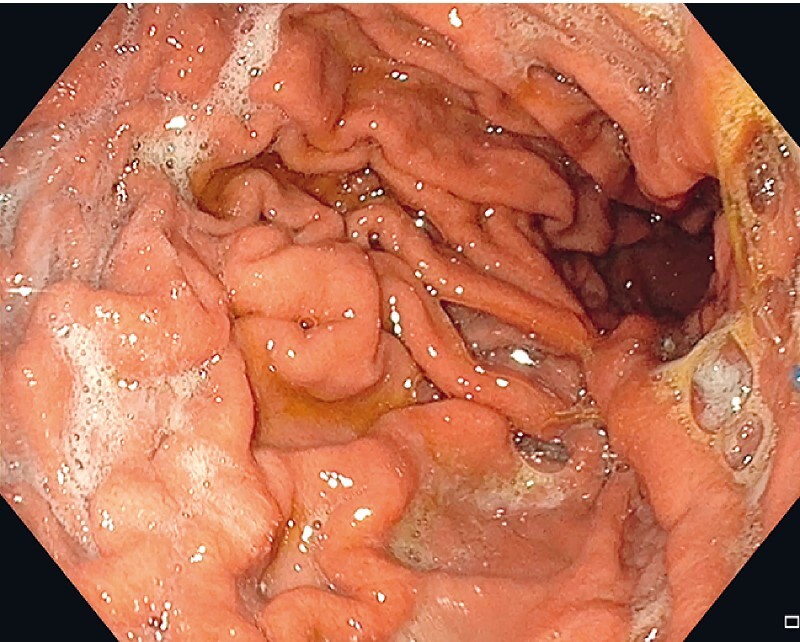
Device introduction via overtube.

Endoscopy_UCTN_Code_TTT_1AO_2AN

Citation Format Endoscopy 2023; 55: E1124–E1125. doi: 10.1055/a-2173-7560 .

## Notes

Endoscopy E-Videos

https://eref.thieme.de/e-videos
*E-Videos* is an open access online section of the journal *Endoscopy* , reporting on interesting cases and new techniques in gastroenterological endoscopy. All papers include a high-quality video and are published with a Creative Commons CC-BY license. Endoscopy E-Videos qualify for HINARI discounts and waivers and eligibility is automatically checked during the submission process. We grant 100% waivers to articles whose corresponding authors are based in Group A countries and 50% waivers to those who are based in Group B countries as classified by Research4Life (see: https://www.research4life.org/access/eligibility/ ). This section has its own submission website at https://mc.manuscriptcentral.com/e-videos
