
JOURNAL INFORMATION
==============================
NLM Title Abbreviation: Endoscopy
Journal ID: Endoscopy
NLM Journal ID: 0215166

Endoscopy

ISSN: 0013-726X
EISSN: 1438-8812

ARTICLE INFORMATION
==============================
PMCID: PMC10965311
PMID: 38531397
DOI: 10.1055/a-2288-8583
Article version: 1
Subjects: Correction

Correction: Cutting-edge novel device in the treatment of obesity

http://orcid.org/0000-0001-6960-1013 Kral Jan 1 2
Selucka Jana 1
Waloszkova Katerina 1
Buzga Marek 3
Spicak Julius 1
Machytka Evzen 1
1 Institute for Clinical and Experimental Medicine, Hepatogastroenterology Department, Prague, Czech Republic
2 Department of Internal Medicine, Second Faculty of Medicine, Charles University, Prague, Czech Republic
3 Department of Physiology and Pathophysiology, Faculty of Medicine, University of Ostrava, Ostrava, Czech Republic
Corresponding author Jan Kral, MD, PhD, MBA Institute for Clinical and Experimental MedicineVidenska 1958/9140 21 Prague 4Czech Republicjan.kral@ikem.cz
Electronic publication date: 2024 Mar 26
Publication date: 2024 Feb
Volume: 56
Issue: 2
First page: C1
Last page: C1

Copyright: The Author(s). This is an open access article published by Thieme under the terms of the Creative Commons Attribution License, permitting unrestricted use, distribution, and reproduction so long as the original work is properly cited. (https://creativecommons.org/licenses/by/4.0/)
Copyright year: 2024
Copyright holder: The Author(s).
License: This is an open-access article distributed under the terms of the Creative Commons Attribution License, which permits unrestricted use, distribution, and reproduction in any medium, provided the original work is properly cited.
License URL: https://creativecommons.org/licenses/by/4.0/

Erratum for: Cutting-edge novel device in the treatment of obesity 56 2 30 1 2024 155 156 Endoscopy 10.1055/a-2209-3374 PMC10827414 38290503

==============================
Correction

Correction: Cutting-edge novel device in the treatment of obesity Kral J, Selucka J, Waloszkova K et al. Cutting-edge novel device in the treatment of obesity. Endoscopy 2023; 55: 155–156, DOI 10.1055/a-2209-3374 . In the above-mentioned article, the statement for the conflict of interest has been corrected. Correct is: The E-Video was supported by Nitinotes, the company that developed this device. This was corrected in the online version on March 21, 2024.

